# [Retracted] Oxidized‑low density lipoprotein accumulates cholesterol esters via the PKCα‑adipophilin‑ACAT1 pathway in RAW264.7 cells

**DOI:** 10.3892/mmr.2023.12965

**Published:** 2023-02-20

**Authors:** Yuncheng Qiao, Dongming Guo, Lei Meng, Qingnan Liu, Xiaohui Liu, Chaoke Tang, Guanghui Yi, Zuo Wang, Weidong Yin, Guoping Tian, Zhonghua Yuan

Mol Med Rep 12: 3599–3606, 2015; DOI: 10.3892/mmr.2015.3864

Following the publication of the above paper, a concerned reader drew to the Editor's attention that the “con” and “ox-LDL” panels in Fig. 1E on p. 3602, and various data panels included in Figs. 3 and 5 on p. 3604, contained apparent anomalies, including what appeared to be matching patternings of cellular data either within the same figure panels or comparing among the data panels.

After having conducted an independent investigation in the Editorial Office, the Editor of *Molecular Medicine Reports* has determined that the above paper should be retracted from the Journal on account of a lack of confidence in the overall authenticity of the data. After having consulted the authors in this regard, they agreed with the decision to retract this paper. The Editor deeply regrets any inconvenience that has been caused to the readership of the Journal.

